# Porcine reproductive and respiratory syndrome virus: web-based interactive tools to support surveillance and control initiatives

**DOI:** 10.1186/s40813-019-0117-x

**Published:** 2019-03-28

**Authors:** Marie-Ève Lambert, Pascal Audet, Benjamin Delisle, Julie Arsenault, Sylvie D’Allaire

**Affiliations:** Laboratoire d’épidémiologie et de médecine porcine (LEMP), Faculty of Veterinary Medicine, Université de Montréal, St. Hyacinthe, Quebec Canada

**Keywords:** PRRS, Sequence, ORF5, Similarity, Epidemiology, Control, Surveillance, ARC&E, Molecular

## Abstract

**Background:**

Control of porcine reproductive and respiratory syndrome (PRRS) represents a tremendous challenge. The trend is now toward managing the disease collectively. In Quebec, area and regional control and elimination (ARC&E) initiatives started in 2011; diagnostic testing, including ORF5 sequencing, and sharing of information among stakeholders are largely promoted. At the provincial level, a data-sharing agreement was signed by Quebec swine practitioners allowing PRRS virus (PRRSV) sequences to be transferred to a database maintained by the Laboratoire d’épidémiologie et de médecine porcine (LEMP-DB). Several interactive tools were developed and are available to veterinarians to allow comparison of PRRSV ORF5 sequences within ARC&E projects or provincially while managing confidentiality issues.

**Results:**

Between January 1st 2010 and December 31st 2018, 4346 PRRSV ORF5 sequences were gathered into the LEMP-DB, involving 1254 sites and 43 practicing veterinarians. Approximately 34% of the submissions were from ARC&E projects. Using a novel web-based sequence comparison tool, each veterinarian has access to information on his/her client sequences and can compare each sequence with 1) commercial vaccine strains, 2) historical samples from the same site, and 3) all sequences submitted to the database over the last 4 years. Newly introduced PRRSV into breeding herds can be monitored using a new sequence comparison tool based on comparison of sequences at the provincial level. Each month, graphs providing the number of introductions per month and the yearly cumulative are updated. Between August 1st 2014 and December 31st 2018, 233 introductions were detected on 180 different breeding sites. Following a data-sharing agreement, veterinarians involved in ARC&E projects have access to an interactive mapping tool to locate pig sites, compare sequence similarity between participating sites and visualize the results on the map.

**Conclusions:**

The structure developed in Quebec to collect, analyse and share sequencing data was efficient to provide useful information to the swine industry at both provincial and regional levels while dealing with confidentiality issues.

## Background

Porcine reproductive and respiratory syndrome (PRRS) is one of the most costly disease for the swine industry due to major reproductive and respiratory problems, it is estimated at 664 million US$ annually in United States [1]. The important heterogeneity observed among PRRS virus (PRRSV) North-American strains, combined with the absence of complete protection following heterologous challenge complicate disease management [2]. Several direct and indirect pathways are involved in transmission of PRRSV between herds, including the introduction of infected animals or semen, transport vehicles, aerosols, flying insects, waterfowl and fomites [3–10]. Controlling the disease is therefore done through avoiding introduction of the virus into a herd. Moreover, since reintroductions of a new strain in a herd are frequent after PRRSV herd elimination process, the trend is now toward managing the disease collectively by implementing area and regional control and elimination (ARC&E) initiatives [11].

In Canada, ARC&E projects are frequent in the Ontario and Quebec provinces which account for the highest number of production sites [12, 13]. In Quebec, ARC&E projects have started in 2011 with eight active projects as of December 2018 [14]. They each include between 35 and 300 production sites and are either located in high or low pig density area. More than 850 production sites are involved in theses ARC&E projects [14]. All initiatives promote communication and sharing of information among stakeholders to better understand PRRS epidemiology in the field with the perspective of implementing effective preventive and control measures. The actions implemented vary according to the ARC&E. They can include increasing external biosecurity, limiting voluntary gilt exposure with herd wild-type strain, vaccinating breeding herds and avoiding introduction of weaners and finishers from outside the zone. In several projects the use of diagnostic testing for PRRSV has increased by requesting sequencing in resurgence of clinical signs or by sampling systematically and periodically herds in absence of clinical signs (e.g. two to four times a year depending on production type and location of the farm).

As a part of these diagnostic procedures, sequencing is considered essential to understand PRRS epidemiology in the field and is largely promoted within ARC&E projects but also provincially [15]. In Quebec, only the presence of genotype 2 (North American type) has been reported [16, 17]. Sequencing helps discriminating between vaccine-like and wild-type strains, and is also useful to indicate new viral introduction from recirculation of an endemic strain. Both scenarios could result in a resurgence of clinical signs, but would require either external or internal biosecurity enhancement, respectively. Furthermore, when a new viral strain is introduced in a herd, sources of contamination need to be rapidly identified to implement appropriate prevention measures. All these urged the development of a structure to collect, analyse and share sequencing data at the herd, ARC&E project and provincial levels.

The objective was to develop interactive tools for swine veterinarians to allow the collection, management and comparison of PRRSV ORF5 sequences within ARC&E projects or provincially while managing confidentiality issues.

## Methods

### Collection and management of data

**Fig. 1:**
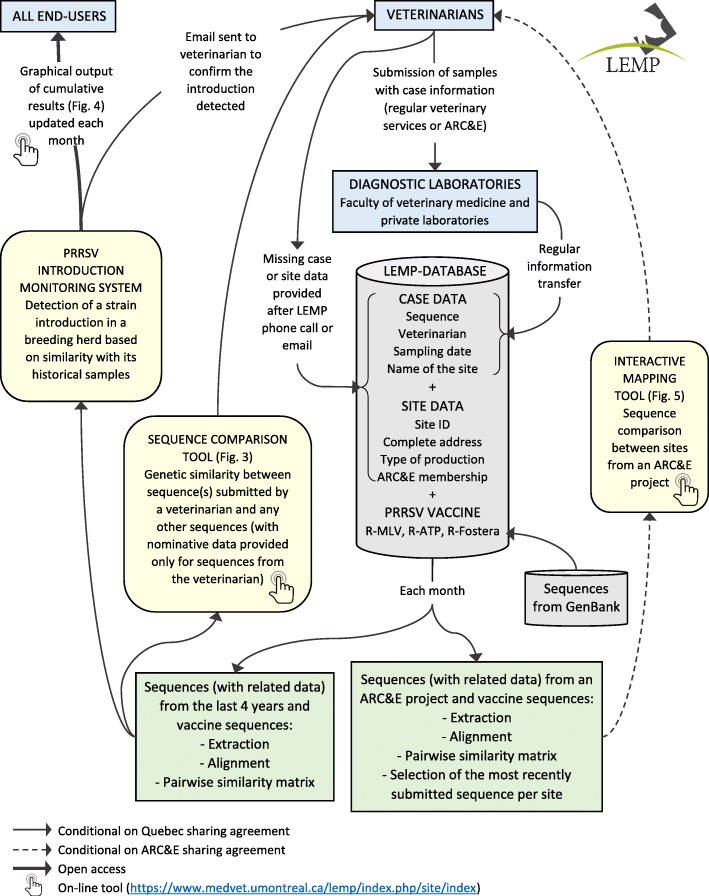
Flowchart of information from the submission of samples by veterinarians to the production of reports from three tools for PRRSV surveillance and control. ARC&E: Area and regional control and elimination; LEMP: Laboratoire d’épidémiologie et de médecine porcine; PRRSV: Porcine reproductive and respiratory syndrome virus

Since 2010, PRRSV ORF5 sequences and information from samples submitted by veterinarians either to the diagnostic laboratory of the Faculty of Veterinary Medicine (FVM) of the Université de Montréal or to other private laboratories from the province of Quebec were transferred upon request from the laboratories and included into the LEMP database (LEMP-DB). Sequences were either collected from regular veterinary services, active surveillance (e.g. ARC&E) or LEMP research projects undertaken in Quebec. Since 2014, a data-sharing agreement signed between swine practitioners and the Université de Montréal, an initiative supported by the Quebec swine veterinary association (Association des Vétérinaires en Industrie Animale-AVIA), has improved the process by allowing the diagnostic laboratories to regularly transfer sequences by email to the LEMP, as well as sequences submitted between January 2010 and September 2014 by batch for each veterinarian. At the time of writing, 97% (37/38) of swine practitioners in the province of Quebec had signed the data-sharing agreement. Figure 1 pictures the flow of information from veterinary submission to their selected diagnostic laboratory to the different tools or reports either at the provincial or ARC&E level.

Sequences obtained from diagnostic laboratories are transferred to the LEMP with additional information limited to sequence identification number, name of the veterinarian, sampling date and the name of the production site (or client). These data are linked to the sequence (fasta format) and registered into an Access relational database (LEMP-DB), for further analyses with phylogenetic software (BioNumerics, Applied Maths Inc., Austin, Texas, USA).

For each sequence submitted, the LEMP also collects additional information on the submitting site from the veterinarian (Fig. 1): correct name of the site, complete address and production type in order to assign the sequence to a specific site ID. A unique ID is attributed by the LEMP to each site which is defined as one or several pig units located at the same address and on the same side of the road. Geographical coordinates of each site ID, an approximate centroid of one or several units, were obtained from Google Earth. When needed, the location was confirmed with the veterinarian.

For each specific ARC&E project, an additional data-sharing agreement was developed and signed by pig and site owners, veterinarians, the project coordinator and the Université de Montréal to allow sharing of nominative data between parties. This agreement allows the LEMP to collect, analyse and report results. Each sequence is assigned or not to an ARC&E project according to the membership of the submitting site.

The dataset also comprises commercial vaccine strain sequences: Ingelvac PRRS® MLV (Boehringer Ingelheim Vetmedica Inc., St. Joseph, Missouri, USA), Ingelvac PRRS® ATP (Boehringer Ingelheim Vetmedica Inc., St. Joseph, Missouri, USA) and Fostera® PRRS (Zoetis, Florham Park, New Jersey, USA), all obtained from GenBank.

### Development of web-based interactive tools for veterinarians

Different interactive tools were developed for veterinarians to provide information on PRRSV either at the provincial level or for specific ARC&E projects. They are available online (in French only) on the LEMP page of the Université de Montréal website (http://www.medvet.umontreal.ca/lemp/).

### PRRS provincial surveillance: Sequence comparison tool

A first tool was designed to help veterinarians easily access the list of all the sequences submitted for their clients for each production site. The veterinarian can select any of the sequences listed and compare its genetic similarity with commercial vaccine strains, historical samples submitted from the same site or other sequences gathered in the LEMP-DB within the last 4 years.

Each month, sequences from the last 4 years are selected from the LEMP-DB along with three reference commercial vaccine strains (R-MLV, R-ATP, R-Fostera). A pairwise alignment was first performed on each pair of sequences (open gap 100%, unit gap 0%) using the Fast Algorithm (minimum match sequences = 1, maximum number of gaps = 3) available in BioNumerics software (version 7.6). Then, a similarity matrix was computed considering a 100% gap penalty, considering the unknown bases and applying a Jukes and Cantor correction. An unweighted pair group method using arithmetic averages (UPGMA) clustering was applied on the similarity matrix. A multiple alignment (open gap 100%, unit gap 0%) was then calculated, as a pairwise alignment of local consensus based on the UPGMA tree in order to obtain a global consensus. Fast algorithm was implemented as previously described. A distance matrix was obtained (100% gap penalty, Kimura evolutionary model) and converted into a similarity matrix using the default display settings. The matrix was exported within a text file (SequenceID1, SequenceID2, genetic similarity %) using a Python script incorporated in Bionumerics. Site data (name of the production site, site ID, complete address, production type) and case data (sampling date, name of the veterinarian) were also extracted from the LEMP-BD within a second text files using a Python script. Both files are then loaded within the web-based sequence comparison tool.

Within the tool, scripts were written to identify vaccine-like sequences: similarity ≥97.5% with one of the commercial vaccine strains (R-ATP, R-MLV, R-Fostera). A cut-off of 97.5% or 98% is often used to declare a sequence as different from another [15, 18, 19]. This cut-off was also chosen as it was shown that the average pairwise similarity among sequences in vaccine-like clusters was 1.1 and 2.4% for MLV and ATP vaccine, respectively, in Eastern Canada [16]. This threshold allows encompassing a majority of vaccine-like sequences while excluding outlier sequences from vaccine-like clusters. An algorithm was developed to extract pairwise genetic similarity of one sequence with other sequences by applying requests on the similarity matrix registered in a MySQL database. A web application was created using a PHP framework to allow secure login, navigation in the sequence register, data sorting and exportation reports generated by the system. The secure login ensures that nominative information is only available for the user (i.e. veterinarian) who had originally submitted the data.

### PRRS provincial surveillance: PRRSV introduction monitoring system

A second tool was designed to monitor the PRRSV introductions in breeding herds at the provincial level using the LEMP-DB and to provide information to the swine industry. Breeding herds included farrowing, farrow-to-wean, farrow-to-finish and gestating gilt operations.

Since August 1st 2014, viral introductions in breeding herds have been identified using PRRSV ORF5 sequences from the LEMP-DB using the following procedure. Monthly**,** each newly submitted sequence from breeding herds was first compared with the three commercial vaccines available for PRRS in Quebec. When similarity was ≥97.5%, the veterinarian was contacted to investigate if the vaccine was used in the herd. If not, the sequence was assigned to a new introduction. Sequences showing < 97.5% pairwise genetic similarity with reference vaccine strains, were further compared with historical samples submitted for the same site to the LEMP-BD during the last 4 years. When a newly submitted sequence had > 92 and < 97.5% pairwise genetic similarity with historical sample(s), the veterinarian was phoned to verify whether the herd had recently undergone a successful eradication process since the submission of the historical sample. If so, it was declared as a new viral introduction. Otherwise, the sequence was not considered as a newly introduced virus. A newly submitted sequence with ≤92% pairwise genetic similarities with historical samples was considered as a new viral introduction [19]. In absence of a historical sample, the LEMP inquired of the veterinarian for PRRS herd clinical history prior to the detection of clinical signs. For herds confirmed to be PRRS naïve by the veterinarian prior to the appearance of the first clinical sign that led to the sampling, the case was considered as a new viral introduction. If not, the case was rejected.

A MySQL web database was developed to register the PRRSV introductions detected. Each month, data are registered in the web database and analysed, graphs are updated and loaded into a specific LEMP web page. These graphs include the number of introduction(s) per month and the yearly cumulative number of introductions. As data are aggregated, graphs are freely accessible on the LEMP web site.

### PRRS ARC&E projects: Interactive mapping tool

The third tool was specifically developed for veterinarians to map pig sites of ARC&E projects, compare sequences submitted by participating sites and estimate Euclidean distance between sites. The information from all sites enrolled in the ARC&E project was transferred in the dataset used by this tool conditional on the signature of a confidentiality agreement by the owner and veterinarian. Once logged using secured access, veterinarians can perform themselves a sequence comparison within each ARC&E project(s) involving his/her client(s), and geographically identify sites having a similar sequence.

Each month, all sequences from sites participating in an ARC&E project and three commercial vaccine strains are selected from the LEMP-DB and the genetic similarity matrix was computed as described previously in section “PRRS provincial surveillance: Sequence comparison tool”. A Python script written in Bionumerics software exports the matrix in a text file (SequenceID1, SequenceID2, genetic similarity %) along with information on both production sites involved in each pairwise comparison: site name, address, geographical coordinates, production type, ownership, attending veterinarian, ARC&E project. This text file is imported in SAS software to filter only the most recent sequence submitted by each production site and to produce a standardized file to be imported by the mapping tool.

For the mapping tool implementation, a geographical information system was developed using open source codes. Data were then projected using Lambert Conic Conform Projection. Vector data from roads and land cover obtained from open sources OpenStreetMap were added to the system. The user interface was developed using a PHP framework**.** Several measurement tools were programmed, all based on open source codes. Pop-up windows were created to visualise nominative information for each site.

The web application developed allows the selection of a pig site to be compared at a selected threshold for pairwise genetic similarity (90 to 100% with 1% increment) at which two sequences are considered similar. Several scripts were programmed in PHP/MySQL to automate the treatment of the requested comparison using the genetic similarity matrix and to present results directly on the geographical map. A tutorial was created to explain the different functionalities to users. A specific web page was designed for each ARC&E project as well as a distinct secured access for each veterinarian according to the participation of their clients.

## Results

**Fig. 2:**
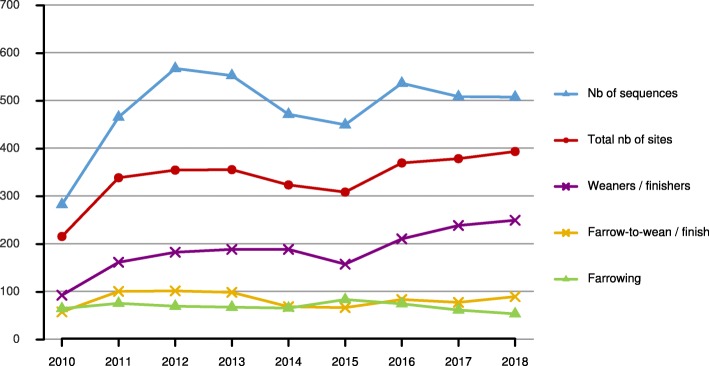
Number of sequences submitted to the LEMP-DB and number of submitting sites according to production type, by year

From January 1st 2010 to December 31st 2018, 4346 PRRSV ORF5 sequences (average of 483 per year) were gathered in the LEMP-DB, involving the participation of 43 swine veterinarians. The median (5th–95th percentiles) annual number of sequences submitted per veterinarian was 10 (1–54); for each veterinarian, only years for which at least one sequence was submitted were considered in this estimation. The number of sequences and number of submitting sites are shown according to year in Fig. 2. The median (5th–95th percentiles) annual number of sequences submitted per site was 1 (1–3); for each site, only years for which at least one sequence was submitted were considered in this estimation. Approximately 34% of the sequences submitted were from sites participating into ARC&E projects.

### Sequence and comparison tool

**Fig. 3:**
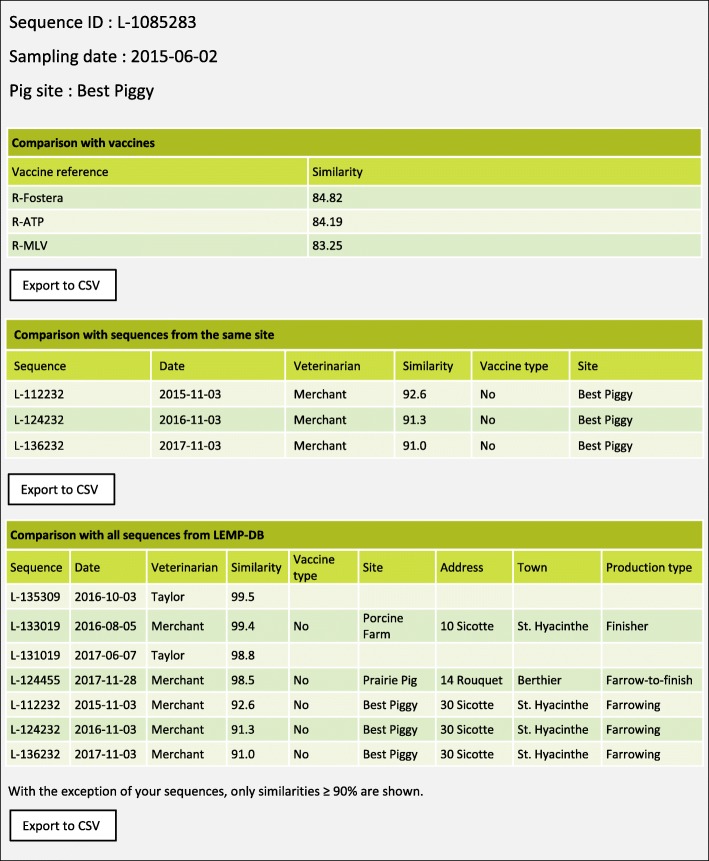
Example of output provided by the sequence comparison tool (fictive data)

Since the launching of interactive tools in 2014, the sequence comparison tool is updated monthly, and swine practitioners are advised by email of each update. Using their password, each veterinarian has access to his/her own sequence list showing the identification number of sequences belonging to his/her clients, sampling date, name of the site as well as the type of strain (wild-type vs. vaccine-like). Once clicking a button, a comparison process based on pairwise genetic similarity (%) can be initiated for each sequence of the list. In a few seconds, results appear for the selected sequence for three types of comparisons (Fig. 3): 1) with commercial vaccine strains, 2) with historical samples submitted from the same site, and 3) with all sequences from the LEMP-DB. For this latter comparison, nominative data (site identification, address, production type) are provided only for sequences submitted by the on-line user. For sequences assigned to another veterinarian, only non-nominative data are provided (sequence identification number, date of sampling, attending veterinarian and genetic similarity). Each report can be exported separately into a csv file to allow inclusion in veterinary medical records.

### PRRSV introduction monitoring system

**Fig. 4:**
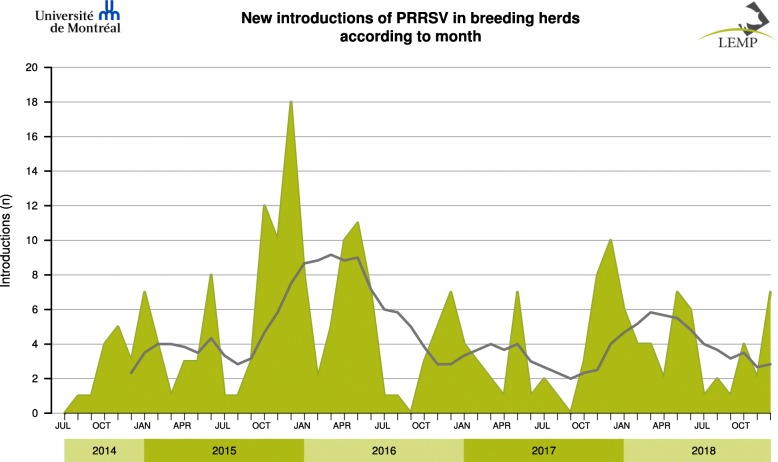
Example of report for PRRSV introduction monitoring system (actual data). The graph illustrates the number of introductions into breeding herds between August 1st 2014 and December 31st 2018. Gray line represents a 6-month moving average

A total of 233 new viral introductions were detected in breeding herds between August 1st 2014 and December 31st 2018. This represented 25% of the total number of sequences submitted by veterinarians for breeding herds for the same period (*n* = 950). The 233 introductions occurred on 180 different breeding sites. Graphs are produced monthly and are freely accessible on LEMP web site (Fig. 4).

### PRRS interactive mapping tool

**Fig. 5:**
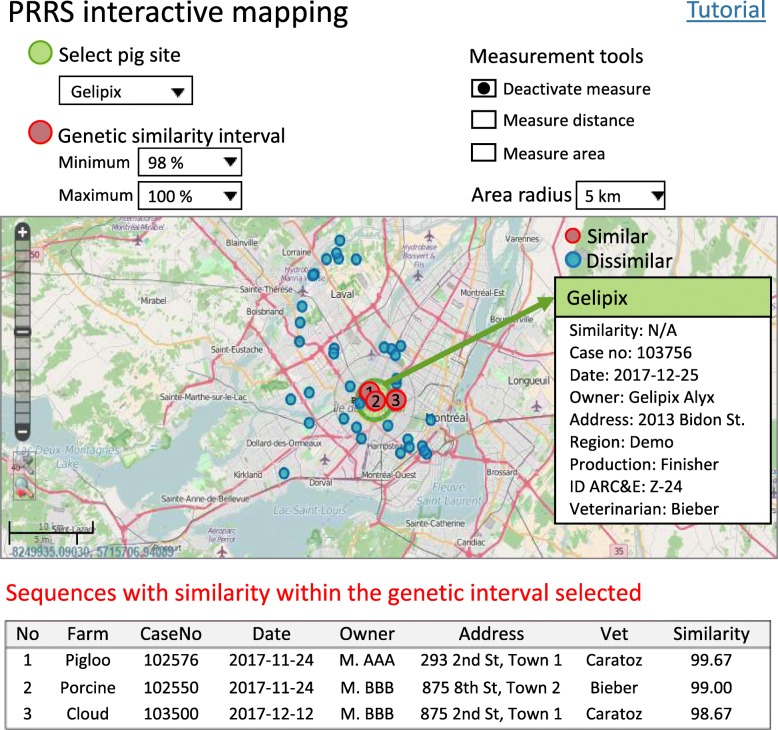
Example of output from the interactive mapping tool (fictive data)

The tool developed to map pig sites participating in an ARC&E project and to compare sequences submitted by participants is illustrated in Fig. 5. Using a drop-down menu, the veterinarian first selects the pig site and a genetic similarity interval to consider two sequences similar (e.g. 98 to 100%) (Fig. 5). Requests are automatically initiated and results appear on the geographical map with green colour marking the selected pig site, red for pig sites having similar sequence, and blue for other sites. Furthermore, each site with a similar sequence (red) is numbered on the map to facilitate the navigation between the map and the summary table (below), which reports all pig sites with a similar sequence with additional site information. At any time, the user can click on a pig site and get a pop-up window reporting case and site information: percentage of similarity observed with the reference pig site, sequence ID, sampling date, site owner, complete address, region, ARC&E ID, and name of the veterinarian. A tutorial explaining the different functionalities was created and is available to all veterinarians. The PRRSV interactive mapping tool is updated each month.

## Discussion

Sequencing of PRRSV is a diagnostic procedure largely used in Quebec with an average of 483 sequences per year between 2010 and 2018. Financial support from the pig industry and provincial government has been provided to promote the use of diagnostic testing, including for sequencing. At the time of writing, the sequence database represented the largest one to be used interactively by field veterinarians within Canadian swine industry. Sequences were from up to 394 pig sites per year, which indicates that approximately 16% of the total number of sites in Quebec (*n* = 2440) performed sequencing at least once annually. It was reported that between 30 to 40% of the sites in Quebec were PRRSV positive to a wild-type strain in 2017 [20], suggesting that several site owners did not submit samples for sequencing even though their herd was positive. Reasons why owners did not use sequencing could include a lack of regular follow-up by veterinary services, financial issues limiting diagnostic testing or a perception that sequencing is not useful. A large number of swine veterinarians (*n* = 43) have submitted at least one sequence over the last 9 years, which suggests that all production types, from independent producers to large pig companies, were using sequencing. Nevertheless the annual number of submissions by veterinarian varied considerably (median, 5th – 95th percentiles: 10, 1–54) and may be influenced by several factors: conviction of usefulness or ability to interpret sequencing results, producer’s decision to submit, number and type of herds attended (production type, herd health status, enrollment in an ARC&E project). Under submission could have led to an underestimation of the number of viral introductions in breeding herds and to a lower number of sequences available for comparison in field investigation. It would be important to determine reasons of under submission to evaluate the representativeness of the sequence database and to adequately promote sequencing.

The on-line access to the list of submitted sequences with client name was in response to veterinary demands. This facilitates the retrieving of specific sequences with their similarity when investigating cases without having to search the entire medical records of his/her clients. This tool is complementary to the information provided in reports from the diagnostic laboratories. The validity of outputs from our comparison tool requires precise and standardized site identification for each sequence as nominative data are included. Therefore, quality of information is crucial.

The PRRSV introduction monitoring system was developed as a more global approach for surveillance at the provincial level. The high prevalence of PRRSV infected herds in some regions precluded using only PRRSV status (positive/negative) as the sole indicator to monitor PRRSV activities within Quebec, especially considering that a large proportion of herds are also vaccinated for PRRSV [20, 21]. The PRRSV introduction monitoring system was the first attempt to use sequencing to document PRRSV introductions in breeding herds. Using a genetic similarity threshold of ≤92% to declare a sequence as new may underestimate the incidence, but it was chosen to be more conservative. Indeed, there is a gray zone for which no consensus is obtained in considering whether two sequences are similar or not. This potential bias was reduced by calling the veterinarian to get further information about eradication process when similarities were > 92 and < 97.5%. The influence of the genetic threshold could be evaluated in the future by a sensitivity analysis also considering time interval between sampling dates. Since tools are based on the ORF5 gene and we cannot preclude that mutations have occurred on other portions of the genome, the number of introductions could also have been underestimated for that reason. On the other hand, the presence of recombination based on ORF5 section was not considered on our whole dataset and this may have resulted in false positive cases of viral introduction. However, the dataset was screened for recombinants in the past and these have been found to be marginal in numbers (17 recombinant sequences out of 2383 ORF5 sequences gathered between 2010 and 2014, unpublished data). Nonetheless, the tool has helped describe the temporal variation of the number of new introductions according to the year, showing that 2015–2016 had the highest number registered. Maintaining this tool on a long-term will be essential to determine the significance of these trends. As these viral introductions were not necessarily associated with clinical signs at the time of sampling, the estimate does not represent exactly the number of PRRS clinical outbreaks. Clinical information would be useful to complement the actual indicator and estimate the economic impact for the swine industry. When updated data on total breeding herds inventory will be available monthly, the percentage of breeding herds with introduction will be added to the existing graphs.

The PRRSV interactive mapping tool is currently offered to all ARC&E projects in Quebec. Using that tool, participating veterinarians can determine if the strain involved in a case of his/her client is also observed on other participating sites of the ARC&E project, or rather consists of a newly detected strain in the area. In the former, it speeds up the case investigation of the source of contamination, since communication and sharing is highly promoted in ARC&E projects. Measurement tools for distance also help to assess if either short or long distance pathways of transmission could be involved. In absence of any similar strain in the ARC&E project, it suggests the introduction of a new strain within the zone, which is important to know as one of the objectives of regional control projects is to limit the number of circulating strains. Only the last submitted sequence is mapped for each site, hence the frequency of diagnostic testing of herds will influence results. To complement that tool with information on historical submission from each site, other reports are also produced by the LEMP monthly. They consist of a phylogenetic tree that includes all sequences submitted for the control project and a similarity matrix matching the tree (not shown), both with nominative data. Whereas the matrix gives the precise genetic similarity observed for any pairs of sequences, the phylogenetic tree provides an overview of relationship among all sequences. At the time of writing, information on the location of PRRS negative sites was not available yet through the tool. Further layers of data will be added in the future to obtain a complete portrayal of participating sites in each ARC&E project.

This article reports the development of web-based interactive tools developed for swine veterinarians to support them in their field investigations, at either the individual herd or ARC&E level. These tools represent an added-value to sequencing, promoting a larger use of sequencing and the maintenance of a database of sequences including meta-data. As a collateral benefit, the information collected allows for further investigations of specific aspect of PRRSV epidemiology, such as the trends in the incidence of introduction or the efficacy of particular collective control measures implemented in ARC&E initiatives. Further studies should be conducted on the efficacy of the tools in supporting PRRSV control through collective actions and potential improvements, with a perspective of favoring the sustainability of the system.

## Conclusion

The structure used to collect, share and analyse sequencing data while dealing with confidentiality issues allowed providing useful information to the swine industry at both provincial and regional levels. The industry would then benefit of these surveillance tools to learn more about PRRS epidemiology and to further evaluate the impact of control initiatives implemented in the field. Tools could be adapted to PRRSV whole genome sequencing once commercially available and experiences learned with PRRSV could also serve for other pathogens.

## Abbreviations

ARC&E: Area and regional control and elimination
LEMP: Laboratoire d’épidémiologie et de médecine porcine
PRRS: Porcine reproductive and respiratory syndrome
PRRSV: Porcine reproductive and respiratory syndrome virus
